# Advances in the Functionalization of Vaccine Delivery Systems: Innovative Strategies and Translational Perspectives

**DOI:** 10.3390/pharmaceutics17050640

**Published:** 2025-05-12

**Authors:** Ingrid Andrêssa de Moura, Anna Jéssica Duarte Silva, Larissa Silva de Macêdo, Karina Mayumi Tani Bezerra de Melo, Lígia Rosa Sales Leal, Benigno Cristofer Flores Espinoza, Maria da Conceição Viana Invenção, Samara Sousa de Pinho, Antonio Carlos de Freitas

**Affiliations:** Laboratory of Molecular Studies and Experimental Therapy—LEMTE, Department of Genetics, Federal University of Pernambuco, Avenida da Engenharia S/N, Recife 50740-600, Pernambuco, Brazil; ingrid.andressa@ufpe.br (I.A.d.M.); anna.jessica@ufpe.br (A.J.D.S.); larissa.smacedo@ufpe.br (L.S.d.M.); karina.mayumi@ufpe.br (K.M.T.B.d.M.); ligia.leal@ufpe.br (L.R.S.L.); benigno.cristofer@ufpe.br (B.C.F.E.); maria.conceicao@ufpe.br (M.d.C.V.I.); samara.pinho@ufpe.br (S.S.d.P.)

**Keywords:** rational design, carrier systems, immunotherapies, vaccines, immune response

## Abstract

The development of effective vaccines requires a rational design that considers the interaction between antigens, their vectors, and the immune system in addition to the activation of pathways that induce a safe and specific immune response. The efficacy of a vaccine formulation depends on the nature of the antigen, the protection offered by the delivery system, the ability to potentiate the immune response, and the precise release of the immunogen. Carrier systems such as lipid nanoparticles, polymers, exosomes, and microorganisms can be functionalized by chemical, physical, or biological methods to generate selective and improved biodistribution profiles. These methods enhance interaction with target cells, thereby improving immunological efficacy. The conjugation of specific ligands or the modification of parameters such as shape, charge, and size of vectors can enhance the specificity, stability, and efficiency of antigen transport to cellular compartments, thereby facilitating a robust immune response. This study examines modifications in vaccine delivery systems, focusing on biomolecules and physicochemical changes that enhance antigen presentation. Additionally, we examine innovative methods, including microneedles, electroporation, and needle-free systems that show potential for enhancing the immune response.

## 1. Introduction

In order for a vaccine to be effective, its rational design must include an understanding of how antigens and their carriers interact with immune cells, and the ability to activate certain pathways that provide a safe and tailored immune response [1,2]. Vaccines, regardless of whether they are intended to prevent or treat diseases, have a high demand to be specific, tolerable, and highly immunogenic [3]. Such requirements lead researchers to a deeper understanding of the immune system and to an improved design for antigens and delivery systems [4].

A prophylactic vaccine antigen must elicit a response that induces memory cells, immunostimulatory molecules, and high-affinity antibodies at the immunological level. This usually implies the use of a T cell-dependent antigen that includes epitopes for both B and T cells, which initiates these immune responses by delivering the necessary signals for activation, recognition, and antigen processing [5]. Therapeutic vaccines, on the other hand, emphasize cellular immune responses, especially those mediated by cytotoxic CD8+ T cells [6]. This calls for critical antigen cross-presentation by dendritic cells and particular strategies directed to enhance T cell activation, typically independent of antibody formation [7]. Interestingly, this is not always the case, and, while less studied, therapeutic vaccines may target antibody formation rather than a powerful cellular response, as in the case of treating opioid-related disorders [8].

However, vaccine formulations include not only the antigen but also the adjuvants and/or carriers needed to enhance the immune response. The capacity of the delivery mechanism to protect the antigen, boost the immune response, and ensure exact administration determines most of the effectiveness of this immunological formulation [9,10]. First, the system should protect the antigen to guarantee its stability and integrity during transportation, therefore preventing early immune system clearance or enzymatic breakdown [11]. This feature was essential for the introduction of nucleic acid vaccines during the COVID-19 pandemic and their subsequent evaluation in clinical trials for cancer, infectious diseases, and autoimmune disorders [12]. Subunit vaccines, on the other hand, can benefit from multifunctional delivery systems that boost immunogenicity while controlling antigenic peptide delivery to the immune system [9,13]. The targeted delivery of the vaccine to specific cells or tissues enables endosomal escape or cross-presentation [14]. These systems may incorporate immunostimulatory drugs, adjuvants, or signaling peptides to enhance antigen absorption and processing by antigen-presenting cells (APCs) [15].

Moreover, to generate selective and optimal biodistribution profiles, carrier systems such as lipid nanoparticles, polymers, dendrimers, exosomes, and microorganisms can be functionalized by chemical, physical, or biological methods [16,17,18,19]. Such approaches promote favorable interactions with target cells, thus enhancing immunological efficacy. The conjugation of certain ligands or modification of characteristics such as shape, charge, and size may improve structural alterations within delivery vectors. These strategies are aimed at improving specificity, stability, and transport efficiency for the vaccine antigen to the cellular compartments where a strong immune response can be elicited [20,21]. Yet, despite these advances, the current literature often examines delivery platforms in isolation or focuses solely on immunological outcomes, neglecting synergies between material design, clinical scalability, and administration challenges.

In this light, this paper aims to contribute to the rational design of next-generation vaccines by analyzing novel tactics that improve the ability of carrier systems to protect, activate, and target antigens, hence boosting adaptive immune responses. Furthermore, we examine the obstacles to translating these technologies and highlight recent breakthroughs in prospective delivery methods.

## 2. Delivery System Functionalization Strategies

Functionalization can be defined as the modification or optimization of carrier systems to increase their efficacy, specificity, and stability for better performance to meet specific requirements in vaccines, imaging, and immunotherapy [22,23,24]. Through the incorporation of targeted ligands and optimization of the physicochemical properties of nanoparticles, researchers have overcome some barriers encountered with traditional formulations and thus expanded their applicability in infectious diseases and oncology therapies [25]. The functionalization of delivery systems has been classified for the purposes of this discussion generally according to the type of system and modifications introduced through different approaches. The subsequent sections will highlight some specific modifications and applications associated with vaccine delivery systems.

### 2.1. Chemical and Biological Functionalization

**Figure 1:**
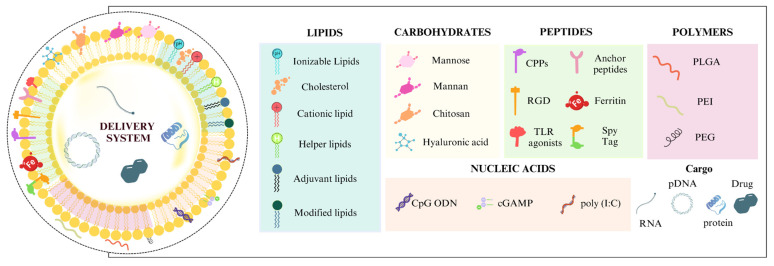
Biomolecules used in the functionalization of vaccine delivery systems. This figure categorizes the main ligands that can be modified or incorporated into delivery systems to improve the efficacy, stability, and targeting of vaccines. The ligands are organized into main groups, including lipids (such as ionizable lipids and cholesterol), carbohydrates (such as mannose and hyaluronic acid), nucleic acids (such as CpG ODN and cGAMP), peptides (such as CPPs and RGD), and polymers (such as PLGA and PEG).

Aiming to raise antigen protection, targeting, and efficacy, chemical and biological functionalization of delivery systems has been thoroughly investigated in vaccine design [16,26]. Whether targeting tumor cells or APCs, delivery systems have been refined in both cancer treatment and vaccinology to raise the specificity, stability, and induction of immune responses [26,27,28]. These interactions can be achieved by incorporating ligands such as carbohydrates, peptides, nucleic acids, lipids, and polymers that influence the interaction between the cell and the delivery system, as demonstrated in Figure 1.

Chemical functionalization modifies delivery systems through covalent bonding, such as the addition of functional groups to nanoparticle surfaces. Examples include amide, disulfide, or thioester linkages, which enhance stability and targeting specificity [28,29,30,31,32]. For instance, the PEGylation of polymers like chitosan improves solubility and extends circulation time [33,34].

Biological functionalization, on the other hand, includes the use of delivery systems with certain properties imparted by components derived from living systems—for example, proteins, peptides, antibodies, or even cells [35]. Antibodies can be coupled to nanoparticles to target specific receptors on immune cells, such as the CD40 receptor of dendritic cells (DCs) [36]. Another approach uses natural or synthetic extracellular vesicles that carry antigens and signaling molecules to induce immune responses [37,38]. In addition, targeting peptides have been commonly adopted, for example, those that bind to the mannose receptor of macrophages, to increase the uptake of vaccines by immune cells [39].

However, a combination of strategies is often utilized to optimize the functionality of delivery systems. The utilization of hybrid systems, such as nanoparticles coated with cell membranes or natural biomolecules, and the use of chemically altered exosomes to enhance biocompatibility and specificity are both effective examples in this field [40,41]. Exosomes tagged with specific ligands, such as antibodies or glycoproteins, can be targeted to dendritic cells, hence enhancing immune activation and lowering off-target toxicity [42]. Conversely, hybrid systems can create safer and superior platforms by combining targeting chemical alterations with the body’s response to what is biological [43].

#### 2.1.1. Functionalization with Carbohydrate Ligands

Carbohydrates, such as mannose, glucan, chitosan, and hyaluronic acid, have been used to improve the immune response as both a ligand and delivery system towards directing and enhancing antigen presentation [44,45,46]. Specialized receptors on the surface of APCs, such as mannose receptors (MRs) and DC-SIGN, help to identify these compounds [47,48,49]. These receptors recognize pathogen-associated molecular patterns (PAMPs), which, given their connection with microbial viability, act as markers for immune cells to identify infections [50].

The simple monosaccharide mannose, in particular, has been widely explored in vaccines as a costimulant with the ability to target and stimulate the maturation of immune cells [48]. Highly expressed in macrophages and DCs, MRs are important for the absorption of mannosylated antigens by phagocytosis and endocytosis, enabling presentation via the major histocompatibility complex (MHC I and II) [44,51]. They initiate the processes associated with cross-presentation and the induction of cellular and humoral immune responses, which are critical for the efficacy of prophylactic and therapeutic vaccines [52,53].

Thus, this saccharide ligand can be chemically conjugated to antigens or delivery systems to improve vaccine efficacy [54]. Techniques such as reversed-phase evaporation and click chemistry reactions have been used to form mannosylated carriers [48,54,55,56,57,58]. In an mRNA vaccine for H1N1, Zhuang et al. (2020) [59] attached mannose to cationic lipids by adding DSPE-PEG-Mannose during the formation of the nanoparticles. This modification resulted in increased antigen expression and both humoral and cellular responses in in vitro and in vivo models. Furthermore, using click chemistry, Nahar et al. (2024) incorporated mannose into liposomes through a Cu(I)-catalyzed azide–alkyne cycloaddition reaction. The mannose/lipopeptide linker improved the immune response against Group A streptococcus (GAS), resulting in increased IgG levels and a balanced Th1/Th2 profile. Additionally, it acted as an independent adjuvant without causing significant inflammation.

On the other hand, Serra et al. (2021) [60] developed binary and ternary delivery systems utilizing mannose-functionalized polyethylenimine (PEI) and octa-arginine peptide (R8) for the delivery of minicircle DNA (mcDNA) vaccines targeting HPV to macrophages. Mannose increased cellular internalization, promoted dendritic cell maturation, and enhanced E7 gene expression. Mannose lipidation and surface anchoring to liposomes also enhanced the efficacy of a cervical cancer vaccine, resulting in reduced tumor growth in animal models [61].

Mannose functionalization also enables targeted vaccination for APC-rich regions, including lymph nodes, where the immune response is started. Apart from enabling the vaccination freeze-drying procedure, the mannose modification of lipid nanoparticles (mLNPs) enhanced the distribution of circRNA-G to lymph nodes, hence encouraging long-lasting immune responses against rabies and SARS-CoV-2 [62]. Nevertheless, functionalization can also be combined with other strategies to further improve vaccine efficacy. For example, Puigmal et al. (2023) [63] developed delivery systems based on the conjugation of allyl-α-D-mannopyranoside (ADM) and mannose-functionalized poly(β-amino esters) (PBAEs) for transdermal vaccination, demonstrating that the addition of mannose increased the transfection of APCs and promoted the maturation of dendritic cells.

Aside from mannose, other carbohydrates such as glucans, chitosan, and mannan have been studied as adjuvants and delivery methods. The mannan, for example, is a polymerized form of mannose that binds to numerous carbohydrate recognition domains and is recognized by several receptors, including MRs, DC-SIGN, and Toll-like receptors (TLRs) [44,54]. In the research conducted by Yu et al. (2022) [64], mannan and loxoribine, which is a TLR7/8 ligand, were conjugated to a *Mycobacterium tuberculosis* fusion protein (CFP10-TB10.4). The conjugation with mannan enhanced vaccine immunogenicity, enhancing APC uptake and generating strong cellular and humoral immune responses, including Th1 and Th2 cytokine production.

Chitosan, a polysaccharide derived from chitin, can bind to different receptors, including TLR2 and TLR4, and is recognized for its mucoadhesive and immunomodulatory properties, being particularly effective in the administration of mucosal vaccines [65,66]. In the study by Li et al. (2024) [33], mannose was conjugated to chitosan-coated poly(lactic-co-glycolic) (PLGA) nanospheres (MCS-PLGA-NPs) to increase the delivery of nucleic acid vaccines against foot-and-mouth disease virus (FMDV). This modification enhanced antigen absorption and immune response in animal models, proving the promise of this approach for mucosal vaccinations.

#### 2.1.2. Functionalization with Peptides and Proteins

Modifications on delivery systems using peptides and proteins are accomplished in numerous ways, including modification with cell-penetrating peptides (CPPs), targeting ligands, anchoring peptides, antibodies, fusion proteins, and TLR ligands, with encouraging outcomes in preclinical and clinical trials [67,68].

Usually consisting of 5 to 30 amino acids, CPPs are a class of small peptides utilized extensively for their capacity to deliver medicinal compounds [67]. Most CPPs have a net positive charge, which lets them interact with hydrophilic and hydrophobic areas as well as negatively charged molecules [69]. This arrangement connects cell membranes and access via several channels, including clathrin-mediated endocytosis, phagocytosis, or even direct penetration [70]. Because of this, CPPs can be used in several ways, including as stand-alone delivery systems [71,72,73,74], as modifiers of pre-existing delivery systems [75,76], or as intermediate linkers that enable antigen adsorption onto nanoparticles [77].

Davoodi et al. (2019) [78] used cell-penetrating peptides (MPG, HR9, CyLoP-1, and LDP-NLS) as stand-alone delivery systems for multiepitope HIV-1 antigens in mice. CPPs formed complexes with DNA, polypeptides, and proteins, which enhanced cellular internalization and immune responses, including antibody production and cytotoxic T lymphocyte (CTL) activation. Also, relying on electrostatic interactions, they can act as intermediaries between the binding of nucleic acids with other delivery systems. Coolen et al. (2019) [77] employed RALA, LAH4, and LAH4-L1 to facilitate the incorporation of mRNA into PLA-NPs. These CPPs formed polyplexes with mRNA, which were adsorbed onto nanoparticles, causing enhancement in the delivery and expression. Alternatively, they may be linked to liposome cholesterol through disulfide bonding or lipidation anchoring [75,76]. Y. Li et al. (2023) [79] functionalized liposomes by binding the CPP CGYKK in a cholesterol molecule in an mRNA vaccine, coding the SARS-CoV-2 spike protein in order to increase stability and delivery efficiency. Finally, modifications in CPP-based delivery systems are another method to improve the response of vaccines. S. Jiang et al. (2025) [80], for example, enhanced the CPP S-Cr9T by incorporating stearyl to optimize DNA vaccine delivery. S-Cr9T establishes stable complexes with plasmid DNA, enhancing antigen expression and immune response in murine models. Dussouillez et al. (2024) [72] also developed LAH4-derived CPPs modified with tyrosines or salicylic acid to enhance mRNA delivery in serum conditions.

Apart from CPPs, peptides specifically targeted for receptors have also been investigated to enhance the distribution of vaccines. Arginine–glycine–aspartic acid (RGD) peptides, which bind to integrins seen on the surface of endothelium and tumor cells, are one example [81]. Targeting nanoparticles to cancer cells with these peptides has been performed as in the work developed by Gan et al. (2018) [82], in which an RGD peptide was attached to virus-like nanoparticles (VLNPs) to provide therapeutic agents to squamous cell carcinoma cells. Furthermore, underlining the possibilities of functionalization in delivery systems for vaccine targeting, the fundamental work by Garinot et al. (2007) [83] produced PLGA nanoparticles functionalized with RGD peptide by photografting to raise the efficacy of oral vaccinations. M cells showed higher absorption of ovalbumin (OVA) nanoparticles by encapsulating them as a model antigen, which also produced a humoral immune response in mice. More recently, for the treatment of HPV16-related cervical cancer, Y. Zhang et al. (2021) [84] developed an RGD peptide-functionalized nanovaccine self-assembled using the RGD-GGG-K18 peptide. The nanovaccine comprised a DNA plasmid encoding the HPV16 E7_49–57_ and the heat shock protein HSP110, therefore enabling targeting to tumor cells expressing αvβ3 integrins. Changing delivery systems with RGD peptides can, however, provide major difficulties since liposomes functionalized with cyclic RGD peptides can cause an acute immune response in mice mediated by IgG and IgM antibodies that results in hypothermia and complement system activation [85].

Because of its biocompatibility and capacity to create stable, monodisperse nanoparticles, self-assembling protein ferritin has also been extensively exploited as a platform for vaccine functionalizing [68]. Most research, however, follows the application of this platform as a stand-alone delivery method whereby antigens are anchored on the surfaces. Aiming to produce protective immune responses against several subtypes of the virus, this method has been investigated in several clinical trials, including studies with ferritin nanoparticle vaccines for influenza (NCT03814720, NCT04579250, and NCT031867). Nevertheless, a single modification can change the ferritin-based delivery mechanism. Based on ferritin nanoparticles functionalized on the surface by ΔN SpyCatcher, W. Wang et al. (2019) [85] devised a therapeutic vaccine with neoantigens. Designed as a means of protein binding, this SpyCatcher-SpyTag system lets antigens conjugate themselves to nanoparticles [86,87]. Through this method, ferritin NPs were functionalized to enable the stable binding of HPV16 E7 antigens and MC38 tumor-derived neoantigens, thereby enhancing cellular immunological response (CTL) and anticancer efficacy [88].

Moreover, functionalizing delivery systems with anchoring peptides has become increasingly important in vaccination research, particularly to enhance antigen presentation and cellular immunological responses, mainly by CTLs, and anticancer efficacy. Concerning cancer immunotherapies, yeasts can be functionalized with peptides to assess antigen binding to the main histocompatibility complex. Using linear peptides fused to the mating protein Aga2 shown on *Saccharomyces cerevisiae*, ref. [89] identified particular ligands for various MHC-II alleles. Conversely, in a mouse model, the anchor protein α-agglutinin was employed in a whole yeast vaccination to exhibit ZIKV B and T cell epitopes, therefore activating CD4+ and CD8+ T cells, neutralizing antibodies, and effector cytokines [90]. Genetically engineered cell membrane nanovesicles have also been applied in this setting. To reverse immunosuppression in the tumor microenvironment, C. Liu et al. (2022) [91] created a nanovaccine combining MHC-I anchoring, anti-PD1 antibody, and B7 costimulatory molecules, which stimulated a strong antitumor immune response. Likewise, Mohammadian Haftcheshmeh et al. (2021) [92] induced a specific immune response against breast cancer by multivalent binding to MHC class II molecules by utilizing immunoliposomes functionalized with the LAG-3 fusion protein and the P5 peptide. Comparatively to soluble immunotherapy, attaching LAG3-Ig to the surface caused DC maturation and more effective antitumor responses in treated animals. W. Wang and Zou et al. (2024) [93] created biomimetic nanovaccines that replicate DCs attached to tumor-associated antigens (TAAs), CC chemokine receptor 7 (CC7), and immunomodulatory compounds in order to modify the TME and increase efficacy.

The modulation of the immune response with ligands that activate the STING or TLR pathway, which is essential to induce innate and adaptive immune responses, has also been explored. Being able to boost the quality of the immune response, even at reduced antigen levels, peptides such as Pam3CSK4 (a TLR2 agonist) encapsulated in PLA nanoparticles modify the immunological response in a vaccination against the hepatitis B virus [94]. This approach has demonstrated the ability to induce a strong and long-lasting humoral immune response with high titers of neutralizing antibodies. Despite this, most studies targeting these ligands are based on other classes of biomolecules such as lipids and nucleic acids, which will be discussed in the following topic.

#### 2.1.3. Functionalization with Lipids, Nucleic Acids and Polymers

Different classes of biomolecules, such as lipids, nucleic acids, and polymers, have proven capable of overcoming challenges related to the stability and effectiveness of delivery systems, in addition to contributing to the effective activation of the immune response. In Table 1, it is possible to observe that each ligand has properties that confer advantages and disadvantages to be considered in the design of vaccine carrier formulations.

Lipid nanoparticles (LNPs) and their derived systems have emerged as notable vectors for lipid functionalization. The composition of LNPs includes various classes of lipids that provide characteristics such as charge, stability, encapsulation, and delivery efficiency to the nanoparticles [106,124]. For instance, the ionizable lipid DLin-MC3-DMA, used in the formulation of the FDA-approved Onpattro^®^, is found as a component in several LNPs [100,125,126] due to its function in encouraging endosomal release and improving nucleic acid distribution. Both in the prophylactic context against SARS-CoV-2 and therapeutically with mRNA-4157 for melanoma, the clinical development of ionizable lipids is also present in the mRNA-LNP vaccines from Pfizer/BioNTech (ALC-0315) and Moderna (SM-102) [100,127,128].

Cholesterol plays a role in membrane fusion and in improving the efficiency of genetic cargo delivery [112]. However, studies have investigated the replacement of cholesterol with other compounds to improve the stability, cellular uptake, and endosomal escape of carrier systems [110,114]. When used to provide plasmid DNA (pDNA) expressing antigens against avian influenza [114], corosolic acid-modified lipid nanoparticles (CLNPs) showed robust humoral and cellular immune responses in animal models. This change showed a similar stabilizing ability and improved cellular penetration, which raised absorption and transfection efficiency. The hydroxyl modification of cholesterol molecules within LNP formulations was also able to improve the delivery of nucleic acid cargo to T cells [129].

Additionally, it has been demonstrated that the incorporation of adjuvant lipids, such as TLR agonists, into LNPs can enhance the immunogenicity of nucleic acid vaccines [21]. In SARS-CoV-2 mRNA-LNP vaccines, replacing the ionizable lipid DLin-MC3-DMA with the TLR7/8 agonist C12-TLRa raised DCs activation, pro-inflammatory cytokines, and adaptive immune responses, including neutralizing antibodies and Th1-type cellular responses [97]. In turn, cationic lipids, such as DOTMA (N-[1-(2,3-dioleoyloxy)propyl]trimethylammonium chloride), DODAC (dioleyl dimethyl ammonium chloride), and DOTAP (1,2-dioleoyloxy-3-trimethylammonium), have adjuvant effects and utilize electrostatic interaction to aid in the encapsulation of nucleic acids in LNPs [105,130]. Common in many phase I and I/II clinical research studies, DOTAP is the most employed cationic lipid (NCT05264974, NCT02065973, NCT04287868, and NCT04573140). Chen et al. (2022) [131] developed a formulation called LNP3, which combines DOTAP, DOPE (1,2-dioleoyl-sn-glycero-3-phosphoethanolamine), and cholesterol, capable of directing mRNA delivery to specific organs and maintaining efficacy even after lyophilization. Auxiliary lipids, such as DOPE, DSPC (2,3-Dioleoyl-glycero-1-phosphocholine), and PEG2000-DMG, play important roles in stabilizing nanoparticles, destabilizing the cell membrane, and in the endosomal escape of nucleic acids [113,123].

The functionalization of delivery systems through the use of nucleic acids such as CpG ODN (a TLR9 agonist) and cGAMP (a STING agonist) has also been researched to further modulate the immune response. For instance, encapsulating these ligands in pH-sensitive liposomes results in robust activation of the STING pathway as well as anticancer responses in melanoma models [118]. Pant et al. (2024) [117] produced the ELI-002 2P vaccine, which targets pancreatic and colorectal cancer. This vaccine was functionalized using CpG ODN that had been modified with an amphiphilic structure (Amph-CpG-7909) to target lymph nodes, resulting in the production of robust T cell responses that are specific for KRAS mutations. Additionally, it has been found to reduce tumor biomarkers and improve relapse-free survival. Tada et al. (2022) [116] also studied how CpG ODN-loaded cationic liposomes can be used to boost immune responses in the mucosa. They found that the release of IL-6 was associated with the formation of antigen-specific IgA. The combination of poly(I:C) (a TLR3 agonist) and MPLA (a TLR4 agonist) in polymeric nanoparticles is another example. These nanoparticles activate innate immune pathways and boost the effectiveness of vaccines [132,133].

Polymers like PLGA, chitosan, and polyethyleneimine (PEI) are often used to improve biocompatibility, cellular uptake, and the controlled release of antigens in delivery systems [134,135]. The biodegradable copolymer PLGA releases therapeutic doses sustainably, while chitosan promotes cellular uptake, and PEI is efficient in cellular internalization but toxic at high concentrations [136,137]. PLGA nanoparticles, for example, were coated on macrophage membranes and modified with PEI to deliver Dendrobium polysaccharides and ovalbumin (OVA), resulting in an increased activation of CD4+ and CD8+ T cells, in addition to elevated levels of cytokines such as TNF-α and IFN-γ [122]. Chitosan-loaded polylactic acid nanoparticles, on the other hand, have been utilized in the formulation of medication delivery as well as preventive vaccine delivery formulations [121,138]. In many cases, surface modification with polyethylene glycol (PEG) is able to improve nanoparticle stability and circulation. However, due to the difficulties associated with the production of anti-PEG antibodies, researchers have been looking for alternatives, such as D-α-tocopherol polyethylene glycol 1000 succinate (TPGS) [139,140,141].

### 2.2. Physical Properties of Vaccine Carriers and Their Immunomodulatory Effects

Vaccine engineering has evolved significantly with the advancement in nanotechnology, allowing the development of more effective and targeted delivery systems [142]. The functionalization of the physical properties of delivery systems, including size, shape, and surface charge, directly influences the interaction with immune system cells, biodistribution, and uptake by target tissues [143]. Studies demonstrate that nanoparticles designed with specific characteristics can modulate the immune response and enhance antigen presentation, promoting the activation of dendritic cells and the differentiation of effector T lymphocytes [144]. Furthermore, it can determine phenomena such as antigen release kinetics, immunogen stability in the biological environment, and the activation of different endocytosis pathways, influencing the magnitude and durability of the immune response [144]. These properties are particularly relevant for targeting specific humoral and cellular responses, impacting the efficacy of prophylactic and therapeutic vaccines.

#### 2.2.1. Size

The size of delivery systems plays a key role, influencing not only the efficiency of uptake but also the type and intensity of the immune response generated, determining the preferential activation of specific cellular pathways and the polarization of the immune response [145]. This process can occur through different mechanisms of endocytosis by which cells internalize molecules and particles from the extracellular environment, and may include phagocytosis, pinocytosis, or receptor-mediated endocytosis [16]. Pinocytosis refers to the ingestion of fluids and molecules, often through small vesicles (generally less than 150 nm in diameter), while phagocytosis involves the capture of larger particles, forming large vesicles called phagosomes (usually greater than 250 nm in diameter) [16,146]. The concept of pinocytosis also encompasses macropinocytosis, clathrin- and caveolin-mediated endocytosis, and endocytosis that occurs independently of these two components [146]. This difference directly impacts antigen presentation and the immune response profile.

In this perspective, Slütter and Jiskoot, 2016 [145], describe the potential for internalization by APCs concerning nanoparticles and microparticles in which micrometric-scale particles can be internalized by receptor-mediated endocytosis and phagocytosis, but their size may limit macropinocytosis. This mechanism is especially relevant for DCs, which use macropinocytosis to capture structures [51], thus favoring the uptake of nanoparticles. Although microparticles are also absorbed by DCs, macrophages capture them more efficiently, favoring presentation via MHC II, while DCs promote presentation via MHC I, which is essential for the activation of CD8+ T cells [147]. Thus, vaccines aimed at stimulating CD8+ T cell responses, such as against tumors or intracellular pathogens, benefit more from nanoparticle formulations. For vaccines targeting humoral responses, the difference between nanoparticles and microparticles is less evident [145].

According to Dong et al. (2021) [144], particles smaller than 200 nm are targeted to the late endosome and can escape into the cytoplasm, favoring presentation via MHC I and stimulating the proliferation of CD8+ T cells, promoting an immune response permissive to the Th1 profile. In contrast, particles larger than 500 nm are processed in early lysosomes and associated with MHC II, favoring the activation of CD4+ T cells and an antibody-mediated Th2 humoral immune response [144,146].

The kinetics of lymphatic drainage are also directly related to the size of the delivery systems. Nanoparticles smaller than 200 nm rapidly reach secondary lymph nodes by passive drainage, while particles between 200 and 500 nm require uptake by DCs and take longer in comparison to reach lymphoid organs. This differentiation has important implications for directing the immune response and modulating the profile of cytokines released [148].

#### 2.2.2. Shape

The shape of the particles also influences their interaction with immune cells. Studies indicate that spheroidal nanoparticles are more efficiently phagocytosed by macrophages compared to cylindrical or discoid nanoparticles, affecting the duration of antigen exposure and activation of the immune response [149,150]. However, particle shape can be optimized to modulate this interaction. For example, elongated particles attach more efficiently to the cell membrane of macrophages, but their internalization is impaired by their size [149,151]. This effect can be explained by the contact angle of the particles with the macrophages, as small angles favor the formation of actin structures for phagocytosis, while larger angles require more energy, making uptake difficult [150].

Niikura et al. (2013). [152] demonstrated that gold rods were more efficiently absorbed by macrophages and DCs compared to spherical and cubic particles of similar dimensions. According to Yue et al. (2011), rod-shaped particles demonstrate rapid internalization by cells, a characteristic that resembles the remarkable cellular infection capacity of the bacillus [153,154]. However, the immune response generated by different formats can vary significantly. For example, spherical nanoparticles and cubic nanoparticles stimulated an increased secretion of pro-inflammatory cytokines despite having a lower rate of cellular internalization [152].

Another relevant factor is the relationship between the specific surface area of the particle and the immune response triggered. It has been observed that particles with a larger surface area per volume can significantly influence the magnitude of the immune response, which is a crucial parameter in the development of vaccine delivery systems [149]. Changing the particle form can also help to avoid fast clearance by the immune system; in this case, rod- or disk-shaped particles showed less immediate recognition by lung macrophages, lowering negative reactions following intravenous injection [150,155].

Therefore, the choice of particle morphology in vaccine delivery systems must be carefully considered, as it affects both the efficiency of cellular uptake and the nature of the immune response generated.

#### 2.2.3. Surface Charge

Surface charge is an essential factor that affects both the stability of nanoparticles and their interaction with biological systems. This property can be modulated by adjusting the proportion of cationic and anionic components during nanoparticle synthesis or by applying coatings with different electrical charges to their surface [150]. Due to the predominant negative charge of cell membranes, positively charged particles have a higher rate of cellular internalization through electrostatic interactions, facilitating uptake by APCs and enhancing the immune response [142,149].

This characteristic is essential for vaccine delivery systems, as more efficient cellular uptake can enhance the immune response. However, the relation between surface charge and immune response can be more complex depending on other factors, such as particle size and shape, as observed in the previous sections [150,153].

Although cationic structures are preferentially chosen to favor APC internalization compared to neutral or negative charges, there is an increased likelihood of the carrier being retained in the interstitium before reaching the lymph nodes [156]. This retention may explain why several ligand-based surface modifications of carriers facilitate uptake by APCs in vitro but do not induce robust immune responses in vivo, as observed by [157]. Thus, optimizing the surface modifications and surface charge of vaccine carriers remains a challenge.

In the context of biodistribution and lymphatic targeting, surface charge also influences the final location of the vaccine delivery systems in the body. Modulation of the charge of RNA-lipoplexes, for example, resulted in changes in the distribution of antigens between the lungs and spleen, impacting the efficacy of the T cell-mediated immune response [155]. Similarly, negatively charged nanoparticles demonstrated greater accumulation in lymph nodes, while positively charged particles favored gene expression and cellular activation in the same lymphoid tissues [150].

Modification of surface charge can also be exploited to improve the immunogenicity of vaccines administered via mucosal routes [158]. Cationic nanoparticles, when used in pulmonary immunizations, resulted in higher antibody titers and greater activation of CD4+ T cells in the draining lymph nodes when compared to anionic particles [149,158]. This effect can be attributed to increased internalization by dendritic cells and the subsequent, more robust stimulation of the adaptive immune system [149].

Thus, the physical functionalization of vaccine delivery systems through surface charge modulation emerges as a promising strategy to optimize cellular absorption, tissue targeting, and immune activation, configuring itself as an essential parameter in the design of new vaccine formulations.

## 3. Functionalized Delivery Systems in Development

### 3.1. Lipid and Polymeric Nanoparticles

LNPs and PNPs are non-viral nanoplatforms recognized as effective carriers for vaccine delivery due to their capacity to regulate substance release, ease of modification, and ability to safeguard antigens and drugs [159,160]. Recent research, including studies by Lozano et al. (2023) [161] and Bezbaruah et al. (2022) [16], emphasizes the potential of modifying these systems. Hence, the functionalization of these carriers with ligands, including carbohydrates, peptides, lipids, and antibodies, has facilitated the creation of more precise and effective vaccines that can elicit robust and enduring immune responses [16,162].

Structurally, LNPs are formed by a combination of ionizable lipids, phospholipids, cholesterol, and PEGylated lipids [106]. This diverse composition protects the components from enzymatic degradation and facilitates endosomal escape after endocytosis, impacting the delivery of biological payloads inside cells [100,128,162]. Due to the easy processing and modification, the conjugation of LNPs with different classes of ligands has been employed to direct these nanoparticles to DCs, increasing antigen uptake and presentation [48,59]. In this way, they have been widely applied in clinical contexts, especially in the development of nucleic acid vaccines against infectious, autoimmune, and neoplastic diseases [163]. The Pfizer-BioNTech (BNT162b2) and Moderna (mRNA-1273) vaccines, for example, used functionalized LNPs that demonstrated high efficacy in inducing protective immunity against SARS-CoV-2 [100,164,165]. In the Comirnaty vaccine, ALC-0315-optimized LNPs effectively transported mRNA to APCs in lymph nodes, hence generating a more robust immune response marked by notable CD8+ cytotoxic T cell activation and expansion superior to unmodified LNPs [166].

However, the selection of lipid composition in carriers can directly affect both the efficacy and delivery of vaccines, imposing a considerable logistical problem, particularly in areas with limited infrastructure. In this regard, the study by Zhang et al. (2023) [167] revealed that the ionizable lipid SM-102 (Moderna) surpasses ALC-0315 in the Pfizer-BioNTech vaccine for intramuscular mRNA injection, antibody generation, and long-term stability. Meulewaeter et al. (2024) [96] also compared the ionizable lipid SM-102 with the C12-200 in addition to exploring the application of alpha-galactosylceramide (αGC) as an adjuvant in mRNA vaccines encapsulated in LNPs. The lipid C12-200 showed increased pro-inflammatory activity and cellular activation. However, it also displayed reactogenicity, including body weight loss, which might restrict its use in some clinical situations. On the other hand, the glycolipid αGC was found to boost the effectiveness of mRNA vaccines, especially the activation of CD8+ T lymphocytes, which are vital for combating cancer and intracellular bacterial infections. Liposomes, a subtype of LNPs, demonstrate a significant activation of the immune response when conjugated to mannose and CPPs [61,76,168,169]. Conversely, in the therapeutic field, Rizwan et al. (2013) [170] modified cubosomes to include TLR agonists, monophosphorylated lipids (MPLs), and chemotherapeutics, exhibiting a better capacity to elicit cellular and humoral responses than liposomes.

Moreover, carrier changes can help to produce formulations suitable for different conditions. PNPs, such as those based on PLGA and natural polymers consisting of chitosan, β-glucans, hyaluronic acid, and dextran, have been used as vaccine delivery systems due to their biodegradability, biocompatibility, and ability to control the release of antigens [28,29]. Functionalized PNPs can encapsulate a variety of biomolecules, including hydrophilic and hydrophobic antigens [171]. Furthermore, to enhance antigen delivery to APCs, these nanoparticles have been conjugated with CPPs, RGD peptides, nucleic acids, or mannose ligands [77,83,172], demonstrating significant effectiveness in the development of orally administered mucosal vaccines [29,77]. Clinically, Ishikawa et al. (2021) [173] examined the safety and immune response of chitosan nanoparticles (CHPs) functionalized with NY-ESO-1 antigen in combination with poly-ICLC adjuvant in patients with esophageal cancer. The findings revealed safety profiles and strong immune responses, highlighting the possibilities of functionalizing polymeric nanoparticles for the development of cancer vaccines. In addition, PNPs linked with CpG markedly enhance the efficacy of antitumor vaccines in animal models. This enhancement is characterized by increased activation of CD8+ T cells, improved accumulation in lymph nodes, and stimulation of the innate immune system [174,175].

### 3.2. Biomimetic Systems

Biomimetic particles are nanoparticles whose surface is integrated or manufactured with biomaterials capable of mimicking biological characteristics and cellular functions [176]. These systems aim to address challenges encountered in various studies, such as preventing drug degradation by enzymes and mitigating undesired immune responses that could compromise their efficacy [177]. To overcome these difficulties, biomimetics emerges as a delivery strategy, mainly due to their characteristics, such as biocompatibility, low toxicity, and ability to overcome physiological barriers [178]. Moreover, biomimetic nanovaccines can evade the reticuloendothelial system, which would otherwise lead to their degradation, thereby extending their circulation time [178,179]. Several cells and their components can be used as biomimetic delivery platforms, such as extracellular vesicles, dendritic cell membranes, tumor cells, and blood cells [178]. In addition, synthetic nanoplatforms such as metal–organic frameworks (MOFs) and metallic nanoparticles (MeNPs) have also been engineered to exhibit biomimetic properties, offering new strategies for vaccine delivery [180,181].

#### 3.2.1. Extracellular Vesicles

Extracellular vesicles, such as exosomes, can originate from different cell types, so their internal content is diverse, carrying proteins, RNAs, DNA, lipids, and other molecules, and mediating intracellular communication [182]. In addition, because they have proteins anchored in their membrane, they can favor endocytosis by target cells and then deliver their internal content [183]. Based on this characteristic and because they are of endogenous origin, exosomes are a beneficial alternative to increase the effectiveness of drug delivery [184]. Thus, biochemical molecules can be incorporated into their internal content, enhancing their versatility for disease treatment [185].

There are several studies addressing the potential of exosomes as carriers, such as Z. Wang et al. (2022) [186], who developed an inhalable vaccine against SARS-CoV-2, where lung cell-derived exosomes (LSCExos) were modified to express the SARS-CoV-2 receptor-binding domain (RBD). The RBD was conjugated through the linker 1,2-distearoyl-sn-glycero-3-phosphoethanolamine (DSPE-PEG-NHS), forming RBD-Exo. Additionally, the use of PEG in cell membranes provides a protective effect under various conditions [187]. It was observed that RBD-Exo has a greater interaction with DCs than with macrophages and B cells. In other experiments, this vaccine, when administered through nebulization, had better results compared to intravenous administration, with higher levels of T cells, IgG, and IgA. In the context of SARS-CoV-2, Shen et al. (2022) [188] altered red blood cell (RBC)-derived exosomes to carry the T cell epitopes of the virus. Thirty-one epitopes presented by HLA-A0201 were conjugated to DSPE-PEG-NHS and subsequently fused with the exosomes. It was observed that the vaccine increased the T cell response in vivo, avoiding any toxic effects.

The study by Huang et al. (2022) [189] created an in situ DC vaccine (HELA-Exos) by adding immunogenic cell death (ICD) inducers (ELANE and Hiltonol) to α-lactalbumin (α-LA)-engineered breast cancer-derived exosomes. This vaccine was tested in mice and human grafts and led to dendritic cell activation and a CD8+ T cell response, suggesting enhanced tumor inhibition in both models. Furthermore, exosome-based vaccines derived from 4T1/Her2 cells have also demonstrated efficacy against HER2-type breast cancer. Yildirim et al. (2021) [190] modified exosomes using CpG ODN and p(I:C), being able to activate antigen-specific primary and memory T cell responses. Furthermore, the vaccine was able to stimulate the secretion of IgG2a and IFNγ antibodies, indicating a Th1 response with tumor regression observed in mice.

Exosomes derived from immune cells, such as DCs (known as Dex), also show promise, being considered immunogenic and capable of prolonging the retention time of molecules [191,192]. Zhu et al. (2022) [193] conjugated Dex with MUC1, a transmembrane glycoprotein present in several types of cancer and which has been evaluated as an antitumor vaccine candidate. It was observed that MUC1-Dex was able to increase the CTL response and decrease tumor growth in mice. Also, exosomes derived from macrophages and T cells are shown to be effective adjuvants, enhancing the action of vaccines and drugs. However, there are not many studies of exosomes derived from other immune cells as vaccine carriers. It was shown that exosomes derived from M1 macrophages administered after vaccination increased the efficacy of a vaccine by creating a pro-inflammatory microenvironment [194]. This study indicates that these exosomes can act as vaccine adjuvants but may also indicate their use as carriers.

#### 3.2.2. Cell Membranes

In addition to extracellular vesicles, cell membranes have also been shown to be a good delivery platform. Generally, they serve as a barrier while participating in processes such as signal transduction, cell adhesion, and immunological recognition, in addition to being biocompatible [195]. For example, the membrane of cancer cells has the advantage of targeting and activating immune cells through antigens on their surface [196]. Regarding the use of cell membranes, Liu et al. (2019) [197] fused DCs and 4T1 breast tumor cells and incorporated the membrane of this fusion into nanoparticles, forming a nanovaccine, described as NP@FM. This system works to present antigens provided by DCs and has the ability to continuously generate tumor antigens, which are subsequently presented to DCs to activate T cells. In this sense, membrane fusion activated more T cells in vitro and prevented tumor growth in vivo, outperforming groups with only dendritic cell and tumor cell membranes. Another study also used a membrane derived from 4T1 tumor cells to encapsulate biodegradable PLGA nanoparticles carrying R837 (a TLR7 agonist) to form the CCMP@R837 nanovaccine [198]. The CCMP@R837 system was shown to be biocompatible and non-toxic in vitro, with greater uptake by bone marrow-derived dendritic cells due to the presence of the cell membrane. It also induced greater activation of these cells (CD86+ and CD80+). In vivo, it reduced tumor growth, increased mouse survival, promoted a long-lasting immune response associated with CTL differentiation, reduced Tregs, and increased memory and effector T cells.

RBC membranes can also be used as a delivery platform, as they are the most abundant cells in the blood and have a long blood circulation time in addition to being able to evade the immune system [199]. Furthermore, when compared to DCs, it is not necessary to stimulate cell differentiation and maturation. Su et al. (2022) [200] developed an RBC-based vaccine with its surface covalently modified with chemotherapy-induced tumor antigens (cAgs) to combine with immune checkpoint blockade (ICB) therapy. In this study, it was observed that cAgs have a better effect on the immune response compared to non-chemotherapy-induced tumor antigens (nAgs) by enriching neoantigens and the presence of heat shock proteins and histones as content. The RBC-cAgs vaccine can also be captured by DCs in addition to stimulating their maturation, increasing T cell production, and creating immunological memory.

Also in this context, nanotoxoids are a biomimetic platform where toxins are complexed in cell membrane nanoparticles, favoring the delivery of virulence factors and a high immune response [201]. Neutrophil membranes, for example, were conjugated with PLGA to the virulence factors secreted by the bacterium *Acinetobacter baumannii*, forming the Neu-NT vaccine [202]. In this study, it was observed that vaccination with Neu-NT promoted the rapid maturation of DCs and B cells, and reduced the production of pro-inflammatory cytokines and anti-*A*. *baumannii* immune response. In this same approach, macrophage membranes were used to capture factors secreted by *Pseudomonas aeruginosa*, forming MΦ-toxoid [203]. It was seen that there was a high humoral response that led to better protection against bacterial infection.

#### 3.2.3. Metal–Organic Frameworks and Metallic Nanoparticles

In addition to cells and extracellular components, MOFs and MeNPs are also classified as biomimetic vaccine delivery platforms [180,181]. MOFs offer high surface area, tunable pore sizes, and the ability to co-deliver antigens and adjuvants, allowing control of antigen release kinetics and promoting prolonged immune stimulation [181,204]. MeNPs are usually derived from gold, iron oxide, and nickel-based systems and have been used to improve antigenic stability, modulate immune activation, and induce robust Th1 and CD8+ T cell responses [180].

Functionalization strategies for MOFs include post-synthetic surface modification with biopolymers such as PEG and hyaluronic acid, lipid bilayers, exosome coatings, or inorganic shells to increase biocompatibility, stability, and targeted delivery [181]. These optimizations improve circulation time and cellular uptake while avoiding opsonization and macrophage clearance [205]. MOFs, such as zeolitic imidazolate framework-8 (ZIF-8), combine metal ions and organic ligands to create porous nanostructures capable of encapsulating biomolecules [206]. ZIF-8 has been shown to protect protein antigens, enable pH-responsive release into endosomal compartments, and intrinsically activate innate immune receptors, including Toll-like receptors 7, 8, and 9, thereby potentiating humoral and cellular immune responses [207].

Furthermore, MeNPs, especially gold NPs (AuNPs), are functionalized in various ways to optimize vaccine delivery and enhance immune response [208]. According to Marques Neto et al. (2017) [180], although the size and shape of MeNPs have little or no effect on the induced innate response, changes in the coating can increase the ability of these molecules to modify immune responses. In this sense, conjugation with oligosaccharides, antigenic peptides, molecular adjuvants, and nucleic acids are strategies used to optimize delivery and increase the immunogenicity of MeNP-based vaccines. For example, Safari et al. (2012) [209] used glycoconjugation-modified AuNPs as carriers for a synthetic conjugate vaccine against *Streptococcus pneumoniae* type 14 that was able to induce specific humoral responses. Furthermore, antigenic peptides, such as the influenza virus M2 protein or *Listeria monocytogenes* epitopes, are conjugated to activate cellular (Th1/Th17) and humoral responses [210,211]. Thus, adjuvants such as poly(I:C) and CpG are also used to increase immunogenicity [152,212]. Furthermore, nucleic acid delivery strategies, such as siRNA against PD-L1 or IDO, are used to modulate immunosuppressive responses [212,213] in addition to coatings such as mannose for targeting dendritic cells [214].

However, despite the inherent advantages, the use of metal carriers also has some disadvantages. Cytotoxicity associated with the release of metal ions, synthesis variability, possible bioaccumulation, long-term toxicity, and concerns regarding stability under physiological conditions limit clinical application [181,204]. Therefore, rational design should focus on optimizing these parameters to reconcile immunological efficacy with the biological safety of these platforms.

### 3.3. Microorganism-Based

Functionalization in microorganism-based delivery systems typically involves changes to the structure, chemical composition, and genetic background of organisms to improve the delivery of antigens, drugs, or genetic material. Various microorganisms, such as bacteria, viruses, archaea, and yeast, have been used to develop novel treatments and vaccines. Nevertheless, there are still obstacles, including safety concerns, manufacturing issues, and clinical translation.

#### 3.3.1. Bacteria as Functionalized Delivery Vehicles

**Figure 2:**
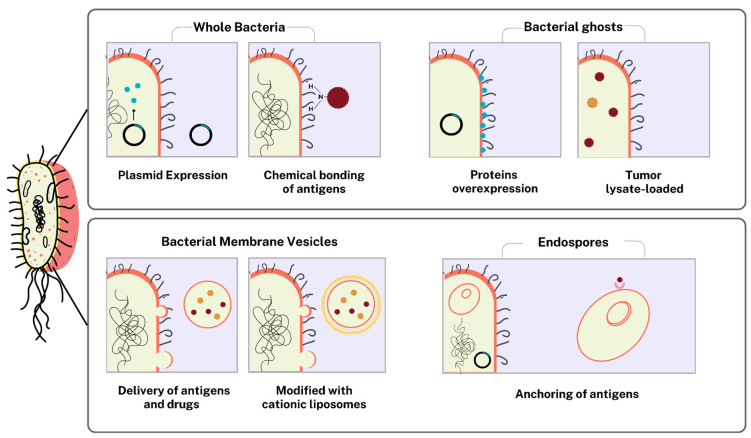
Strategies for bacterial functionalization of vaccine delivery systems. Whole bacteria can be modified by expressing plasmids that produce antigens or by chemically conjugating antigens to their surfaces. Bacterial cell derivatives, such as bacterial ghosts, can be engineered to overexpress immunogenic proteins or incorporate tumor lysate, whereas endospores can anchor antigens. Bacterial membrane vesicles can be used to deliver antigens and drugs, and their functionality can be improved by adding cationic liposomes.

Whole bacteria can be used as models for the delivery of protein antigens, genomic material, or drugs, as they allow the biological function to be maintained, increasing the efficacy of the strategy as therapy [215]. In a prophylactic approach, Ding et al. (2024) [216] used *Escherichia coli* as a vector in the administration of multiepitope structures against the rabies virus (RABV) based on the expression of the glycoprotein and viral nucleocapsid introduced into the pCDNA3.1(−) vector. After oral vaccination in mice, a decrease in viral load and an increase in the presence of neutralizing antibodies against the virus were observed. On the other hand, bacterial carriers can be chemically conjugated to present tumor antigens on their surfaces. Xie et al. (2024) [217], for example, used the anaerobic bacteria *Veillonella parvula* functionalized with the drug DMnSH, which was conjugated to the membrane’s primary amino groups. The system’s intravenous administration specifically targets the hypoxic regions of tumors, allowing drug entry into tumor cells, resulting in cell death and immune response activation in murine models with 4T1 tumors. However, structures generated from this organism have been sought as means of ensuring safe delivery and controlled immune activation due to their high immunogenicity and difficulties associated with safety and immune control. In this regard, bacterial ghosts (BGs) and bacterial outer membrane vesicles (BMVs) have come to the fore as methods of vaccine delivery (Figure 2).

##### Bacterial Ghosts and Bacterial Membrane Vesicles

The use of empty BG bacteria serves as a new vaccine strategy compared to models of inactivated microorganisms or protein subunits; they can strongly stimulate the immune system and can also be used as delivery models. The use of the Lysis E gene of bacteriophage PhiX147 allows the generation of BG models of *Brucella abortus* without altering its antigenic surface. These empty structures provide suitable protection for the immune system after vaccination [218,219]. Ji et al. (2020) [220] used the BG of *Vibrio parahemolyticus* and the plasmid vector pJSL24 to overexpress the surface proteins VP1667 and VP2369 in a veterinary vaccine. Vaccination by the subcutaneous route in zebrafish increased the innate immune response and survival against the microorganism. Furthermore, Hoseini Shahidi et al. (2019) [221] produced BGs from *E*. *coli* O2:K1 transformed with the pmET32b vector, where chickens vaccinated subcutaneously generated an immune response capable of preventing the sequelae produced by colibacillosis. In studies directed against human diseases, Miri et al. (2015) [222] used the *E*. *coli* BG to facilitate the delivery of the chimeric protein gene Core-NS3 of the Hepatitis C virus in murine macrophages RAW 264.7 for its expression, proposing a new transfection model that can be used in the production of effective prophylactic vaccines. Dobrovolskienė et al. (2018) [223] demonstrated that the use of BG loaded with tumor lysate, LPS, and IN-γ induces the ex vivo maturation of dendritic cells with the capacity to promote and direct the activation and production of antitumor cytotoxic CD8+ T lymphocytes.

Finally, the engineering of bacterial membrane vesicles (BMVs) is a new strategy used for the treatment of cancer diseases and the elimination of drug-resistant organisms. Although the reports on BMVs that are currently available were conducted using murine models, they bring new meaning and perspective for the development of more models and clinical trials. Niu et al. (2025) [224] used BMV obtained from *Rhodobacter sphaeroides* for the encapsulation and delivery of copper metal–organic structures with the aim of activating apoptosis in tumor cells. The vesicles were introduced intravenously into mice inoculated with 4T1 breast tumors. Following photothermal treatment (808 nm) generated a strong antitumor response, increased apoptosis, and reduced tumor. N. Li et al. (2024) [225] produced VMB from *E*. *coli* functionalized with D-propargylglycine and azide (IR820) and showed it to accumulate in tumor tissues. After intravenous vaccination and phototherapy treatment in murine models, the vaccine promoted DC maturation, improved T cell infiltration, and polarized M2 to M1 macrophages for the elimination of triple-negative breast cancer (TNBC) tumors. In a similar strategy, X. Liu et al. (2023) [226] chose to produce a VMB from *Fusobacterium nucleatum*, loading them with metronidazole and iron metal–organic structures. After intravenous vaccination in murine models, tumor reduction, elimination of the colonizing bacteria *F*. *nucleatum*, DC maturation, and T cell infiltration into the tumor tissue were observed. Finally, Jia et al. (2024) [227], in a new strategy against drug-resistant *Acinetobacter baumannii* and *Pseudomonas aeruginosa* bacteria, proposed the use of CRISPR/Cas9 loaded by electroporation in the VMB of *E*. *coli* modified with cationic liposomes. The structures used as a vaccine model did not generate changes in tissues or blood cells but had high specificity in eliminating bacteria.

##### Spores as Delivery Vehicles

Bacterial endospores can also be used for the delivery of antitumor drugs or vaccine antigens. They are based on the ability to recognize hypoxic environments and, after germination, promote the lysis of tumor tissue [215]. Heap et al. (2014) [228] genetically modified *Clostridium sporogenes* to express the nitroreductase from *Neisseria meningitidis* with the ability to degrade the prodrug CB1945 to form 4-hydroxylamine (a highly toxic compound). After subcutaneous vaccination in murine models grafted with human colorectal carcinoma cells HCT116, it was shown to be able to eliminate tumor tissue in the animals.

Spore proteins facilitate the anchoring of antigens on their surface, subsequently utilized as vaccines to elicit an active immune response against targeted microorganisms in murine models. Mauriello et al. (2004) [229] anchored the antigen from the C fragment of the *Clostridium tetani* toxin and the B subunit of the heat-labile toxin of *E*. *coli* on the surface of the *Bacillus subtilis* spore, promoting the production of IgG and IgA antibodies. Tian et al. (2024) [230] managed to develop the chimeric gene CotC-Linker-COE to anchor the neutralizing epitope CO-26K of the glycoprotein of the porcine epidemic diarrhea virus (PEDV) on the surface of the *Bacillus subtilis* spore. Immunization through the oral route allowed a substantial humoral response. Min et al. (2024) [231], with the help of the CotB protein, managed to anchor the GP5 glycoproteins of the European and American genotypes of the porcine reproductive and respiratory syndrome virus (PRRSV) on the surface of the *B*. *subtilis* DB104 spore. In the evaluation after subcutaneous vaccination in chickens, high levels of IgG and IgA antibodies were observed. Finally, Sibley et al. (2014) [232] generated recombinant *B*. *subtilis* spores using the chimeric CotB-MPT64 gene, which allowed the MPT64 protein of *Mycobacterium tuberculosis* to be anchored on the surface of the spore, facilitating its recognition by the immune system, reducing the bacterial load in the lungs, and activating Th1 lymphocytes. In the same sense, Das et al. (2016) [233] used the Ag85B and CFP10 antigens of *M*. *tuberculosis* anchored on the surface of the *B*. *subtilis* spore with the help of the CotC protein. Immediately after vaccination via the nasal route, they achieved the production of IgG and IgA.

#### 3.3.2. Phages, Virosomes and Archaeosomes

Among viral systems, phage vaccines use bacteriophages that have their genetic material (DNA or RNA) covered by capsid proteins, which infect and replicate in bacteria and archaea but have no direct effects against eukaryotes [234,235,236]. As a delivery platform, they have stability, biocompatibility, low cost, and ease of production [237,238]. In addition, their intrinsic natural adjuvant properties, nanoscale size, and multivalency capacity have been widely exploited to improve the stability and immunogenicity of vaccine antigens [234,239,240].

Morphologically, they can be classified into filamentous phages, including M13, fd, and f1; icosahedral phages with tails, such as the lytic T4 and T7 and the temperate λ; and icosahedral phages without tails, such as Qβ and MS2 [241]. This variety of shapes and formats allows the selection and functionalization of these vectors to improve lymphatic turnover, capture by APCs, and immunogenicity profile. Recently, published reviews have focused on modifications in these systems and the implications of these alterations for different strategies, from vaccines and immunotherapy to imaging tests [234,238,242,243]. In this sense, Wang et al. (2024) [238] discuss non-covalent modifications, including electrostatic interactions with transporters and ligands, covalent bioconjugation modifications, and genetic modification, as methods to increase the efficacy of phages as delivery vehicles.

In general, the display of antigens on the capsid surface, chemical conjugation with adjuvants or ligands, and integration with hybrid systems, in addition to structural modifications, are used to overcome biological barriers [234,238,243]. In this way, antigen display can occur by the genetic fusion of tumor or pathogen epitopes to phage structural proteins, such as pIII and pVIII (in M13), HOC and SOC (in T4), or gpD (in λ) [238,244,245,246,247,248,249]. This approach promotes a repetitive and ordered presentation of antigens, resulting in the activation of APCs and induction of Th1/Th2 responses [242]. For instance, the T4-Hoc/F1tv vaccine demonstrated complete protection against *Yersinia pestis* in animal models, and research is currently underway for *Bacillus anthracis* [250,251]. Also, the M13-pVIII with GM-CSF phage has been shown to elicit immune antitumor responses against colorectal cancer in BALB/c mice [252,253].

Chemical conjugation allows the coupling of targeting ligands, such as the RGD-4C peptide, or immunopotentiating adjuvants through NHS-ester amidation reactions or click chemistry [238,254,255]. This functionalization improves cell targeting and enhances immune activation, as evidenced by the use of M13-PEI for the electrostatic adsorption of vaccine antigens [256]. Davenport et al. (2022) [257], for example, functionalized lambda phage PLPs by chemically conjugating coronaviral RBDs to the gpD(S42C) protein. This method produced a multivalent vaccine that elicited strong and enduring neutralizing responses and was capable of providing protection against viral challenges in mice. In addition, systems such as AAVP, a hybrid between phage M13 and AAV, have demonstrated efficacy in delivering therapeutic genes such as TNFα in melanoma models, resulting in tumor regression [258,259,260]. In mRNA vaccines, icosahedral phages such as MS2 have been used to increase the protection of the genetic material against nucleases and promote specific immune responses [261]. Moreover, to prolong the systemic half-life and reduce clearance, structural modifications such as PEGylation or coating with cationic lipids have been applied to increase circulating stability [262,263]. However, issues such as safety, possible interference with the microbiota, and the generation of antibodies against the carrier have limited their clinical application [238,264].

On the other hand, virosomes are safe, non-replicating vesicles based on viral envelopes that retain surface proteins from the influenza virus [265]. Proteins such as hemagglutinin and neuraminidase present in virosomes facilitate the transport and diffusion through the membrane of APCs for antigen processing and presentation. The modification of antigen presentation or incorporation of molecules for immune response are the main forms of functionalizing these systems [266]. Epaxal^®^ and Inflexal^®^ V are examples of virosome vaccines approved for clinical use. In Inflexal^®^ V, functionalization using influenza virus hemagglutinin induces a protective immune response against several strains of the virus, while Epaxal^®^ induces a strong humoral immune response through virosomes functionalized with hepatitis A virus antigens [267,268]. Although these vaccines are already in use, additional studies on the internal and surface functionalization of virosomes are underway to scale up for cancer and infectious disease therapy [269,270].

Moreover, archaeosomes are polar ether lipid structures that consist of glycolipids from Archaea. These vesicles function essentially as immunopotentiators for antigens on antigen-presenting cells, guiding the processing of antigens to elicit Th1, Th2, CD4+, or CD8+ lymphocyte responses while also enhancing immunological memory following treatment [271,272]. McCluskie et al. (2017) [273] sulfated *H*. *salinarum* archaeosomes with 6′-HSO_3_-D-Man-p-α-1,2-D-Glc-p-α-1,1-archeol derived from *H*. *volcanii*. The immune responses were characterized by IFN-γ production and CTL activation that were observed upon vaccination with the ovalbumin-loaded system. In vitro studies by Vidakovic et al. (2024) [274] proved that archaeosomes derived from *S*. *acidocaldarius* can successfully load calcein and insulin in target cells. The same authors have further shown that this system can work as an oral model vaccine since it can survive harsh conditions like gastric fluid.

#### 3.3.3. Yeasts as Functionalized Delivery Vehicles

Yeasts are unicellular fungal organisms that have also been explored for their potential as delivery systems [275]. They possess characteristics that are considered relevant for the selection of a delivery platform, such as thermostability, the ability for repeated administration, non-cytotoxicity, the possibility of oral administration, and a generally recognized as safe (GRAS) status by the FDA [19,276,277]. Yeasts are organisms widely used in biotechnology due to their relatively short reproductive cycle, low cost of large-scale expansion, and average cell size between 2 and 4 μm. In addition, elements of their cell wall interact with immune cells, thus improving the immunogenicity of vaccines [275,277,278,279,280].

The functionalizing strategies of this microorganism primarily concentrate on exploring the components of its cell wall, which is mainly composed of β-1,6-glucan and β-1,3-glucan as well as chitin, mannans, and other saccharides [281]. In the context of antigen presentation, glucans interact with pattern recognition receptors (PRRs) present in macrophages, dendritic cells, and neutrophils. This opens opportunities to investigate several possibilities for using the recognition mechanisms of these cell wall components. Among them, β-1,3-glucan stands out for interacting through PAMPs with the Dectin-1 receptor, which is highly expressed on the myeloid lymphocytes, thereby enhancing immunogenicity [275,282,283]. The Dectin-1-β-1,3-glucan interaction also generates a strong inflammatory response to stimulate these phagocytes. The mannans in the cell wall are also capable of interacting with other PRRs, such as DC-specific intercellular adhesion molecules 3 (DC-SIGN) and Dectin-2, thereby triggering a pro-inflammatory Th1/Th17 profile response [275,284,285,286].

**Figure 3:**
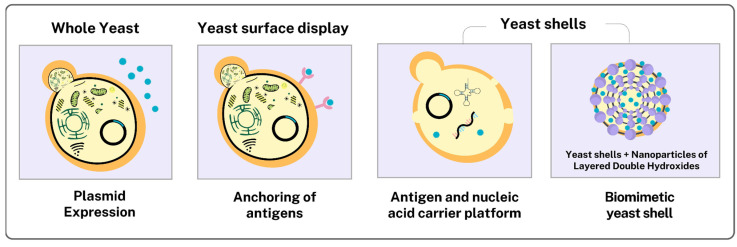
Main strategies for using yeast as antigen delivery platforms. The first box shows the genetically modified intact yeasts that express intracellular antigens. The second represents the yeast surface display, which involves the anchoring of antigenic proteins to the yeast cell wall. The third box shows the capsules created by the extraction of intracellular yeast components, forming yeast shells that are utilized for the transport of nucleic acids and antigenic proteins. Biomimetic yeast shells are yeast capsules that have been functionalized with nanoparticles to enhance surface antigen delivery and modulate the immune response.

Vaccines that use yeasts as carriers for their antigens are based on three main approaches: whole yeast vaccines (WYVs), surface display vaccines (YSD), and yeast capsule vaccines (YS) (Figure 3). WYVs are based on yeast inactivation, either through heat treatment or freeze-drying, to prevent contamination risks. This heat treatment does not inhibit the antigen delivery capacity or the induction of both humoral and cellular immune responses without affecting the yeast’s ability to express the vaccine antigen inside. This strategy allows for the delivery of molecules such as DNA and RNAs (including shRNA), as well as proteins [275,279,287,288,289]. Tests with heterologous protein indicated that the yeast can be stored at room temperature, remaining stable for over a year at 23 to 25 °C, and can also be stored between 2 and 8 °C. The latter temperature range showed the highest quantity of heterologous proteins [275,279,290]. The most commonly used species in this strategy are *Saccharomyces cerevisiae* and *Pichia pastoris*. Both have fully sequenced genomes, enabling multiple genetic engineering approaches. *S*. *cerevisiae* is the oldest species to be explored and sequenced, capable of developing auxotrophic strains, compatible with multiple plasmid vectors, and possessing a high capacity for the mannosylation of recombinant proteins. However, its protein expression rate is lower than that of *P. pastoris*, which has a higher concentration of glucans (potentially enhancing immunogenicity through interaction with PRRs) and an endogenous promoter induced by methanol that triggers a strong expression of recombinant proteins [275,291,292,293].

Vaccines in preclinical study stages demonstrate a variety of possibilities for administering WYVs: subcutaneous injection, such as the vaccine with the L1 gene from human papillomavirus, which used *P. pastoris* as a carrier, and the vaccine with Ag85B from *Mycobacterium tuberculosis*, which used *S. cerevisiae* as a carrier [292,294]. A phase 1 intramuscular injection study used the Apical Membrane Antigen-1 (AMA-1) from *Plasmodium falciparum* carried by *P. pastoris* [295]; an intraperitoneal injection study used the Exocellular glycoprotein (gp43) from *Paracoccidioides brasiliensis* carried by *S. cerevisiae* [296]; oral administration has been explored in vaccines such as those containing the Tp1, Tp2, and Tp9 antigens from *Theileria parva* and the hemagglutinin (HA) antigen from the H5N1 influenza virus, both carried by *S. cerevisiae* [297,298]. In addition to the diversity of administration routes, it has already been observed that this vaccine strategy can affect the expression of gene cascades associated with immune responses (IL-6, IL-10, IL-12, and TNF-α). This was demonstrated through the oral administration of recombinant yeast with shRNA containing the CD40 sequence, which was capable of repressing CD40 in vivo [289].

The surface of yeast is also widely explored due to the large quantity of glucans and other structures that enable cellular interaction. In this context, another strategy used is yeast surface display (YSD), which involves anchoring the antigenic protein to the yeast surface using fusion methods that allow the antigen to be displayed on the surface. The most commonly used anchoring methods are based on the C-terminal glycosyl-phosphatidylinositol (GPI), anchored to the wall and linked to agglutinins that present the antigenic protein. The N-terminus-free display method refers to the binding of α-agglutinin through a covalent bond between its Aga1 and Aga2 subunits (linked by disulfide bonds) and the GPI in the cell wall. The C-terminus-free display method, on the other hand, is based on the binding of the C-terminal half of α-agglutinin to glucans and the GPI in the cell wall [275,299].

This anchoring strategy on the surface requires that the gene inserted into the plasmid vector be accompanied by a secretion signal sequence-encoding gene, the 3′-half of the α-agglutinin gene, and finally the GPI-anchored attachment region. After cellular transformation, these genes are transcribed, and their proteins are synthesized. These proteins will aid in confirming the synthesis of the protein of interest, the formation of α-agglutinin, and the GPI, which will be anchored to the cell wall and linked to the protein, respectively, thus exposing it on the surface for presentation to the immune system cells. In addition to agglutinins, other proteins such as Pir, Cwp2, and Flo1 can also be used for anchoring [275]. Marshall et al. (2013) [300] were able to achieve chemical functionalization by anchoring a protein called intein to the yeast surface conjugated with single-chain antibodies. To later release these proteins, different chemical compounds were used, including 2-mercaptoethanesulfonic acid (MESNA) and hydrazine azide.

A functionalization strategy that has recently been explored is the formation of capsules, either at the micrometer level (microcapsules) or the nanometer level (nanocapsules), with sizes varying according to the treatment used to obtain the capsules. The formation of capsules from whole yeast is carried out by growing the yeast, which is then subjected to an acidic–alkaline treatment with NaOH and HCl to remove intracellular components, followed by washes with distilled water and alcohol to remove residual components, forming glucan particles (GPs), also referred to as yeast shells (YSs) [301]. Different approaches are also explored to create a trapping mechanism to retain the antigen inside the capsules, such as the use of PEI with tRNA, which works by forming a core to encapsulate DNA [302]; the mouse serum albumin (MSA)–yeast RNA (yRNA) complex [303]; a thermostable silica cage [304]; and nickel nanoparticles [305]. Soto et al. (2023) [305] tested different concentrations of nickel to carry the Cda2 protein. These studies demonstrated that this approach achieved an encapsulation efficiency of between 60 and 80%, showed the ability to be phagocytosed by macrophages, and, when tested in vivo, exhibited a protective effect. However, this effect was lower than that of the control group, which used yRNA. Additionally, there is the possibility of carrying antigens on the surface of the capsule using nanoparticles of Layered Double Hydroxides (LDHs). These nanoparticles, through electrostatic forces along the entire surface, facilitate antigen presentation and delivery [275,306]. This approach was able to stimulate Th1 and Th17 profile responses in the study by Liu et al. [306].

Most studies focused on the use of capsules for vaccine development, typically using RNA molecules as the type of antigen, such as siRNA and shRNA. Cohen et al. [307] loaded siRNA against osteopontin into GPs and successfully silenced this gene in obese mice, improving the metabolic phenotype through intraperitoneal injection. Aoudi et al. [308] used siRNA to silence Map4k4, which induced the suppression of TNF-α and IL-1β through oral administration. Zhang et al. (2021) [309] used shRNA targeting IL-1β carried by capsules, which induced the downregulation of the inflammatory response in mice, also through oral administration. There are also studies that utilize GPs for drug delivery in cancer therapy, demonstrating that they are a safe vaccine carrier without causing cytotoxicity. These capsules are capable of transporting the drug without the risk of degradation by enzymes [310] and therapy against type 1 diabetes mellitus, where microcapsules are used to carry insulin [311].

A recent strategy that has been explored in veterinary medicine, with the potential for expansion to human use, involves utilizing only modified yeast wall particles, such as sulfonated β-1,3-1,6-glucans. This is an interesting approach because it targets components with a higher interaction with the receptors of APCs. The study by Wang et al. (2014) [312] used these sulfonated glucans with glyceraldehyde-3-phosphate dehydrogenase (rGAPDH) in chickens. In both animals, the results were promising, as an increase in IL-2, IFN-γ, antibody production, transcription of immunomodulators, and survival rates was observed.

## 4. Routes of Vaccine Administration: Traditional Methods and Innovations

For subunit vaccines, which rely on phagocytosis for antigen presentation, and nucleic acid-based vaccines, which utilize intracellular processes to present antigens via MHC pathways, the route of administration and delivery system design are complementary strategies [10,313]. While the carrier formulation addresses challenges such as low immunogenicity and the inability to efficiently target and reach the intracellular compartment of APCs, the route of administration determines how and where the vaccine will interact with the immune system. Furthermore, the choice of appropriate routes of administration to deliver vaccine antigens to the immune system should be considered with respect to the expected response and clinical practice, whether prophylactic or therapeutic in nature [314,315].

**Figure 4:**
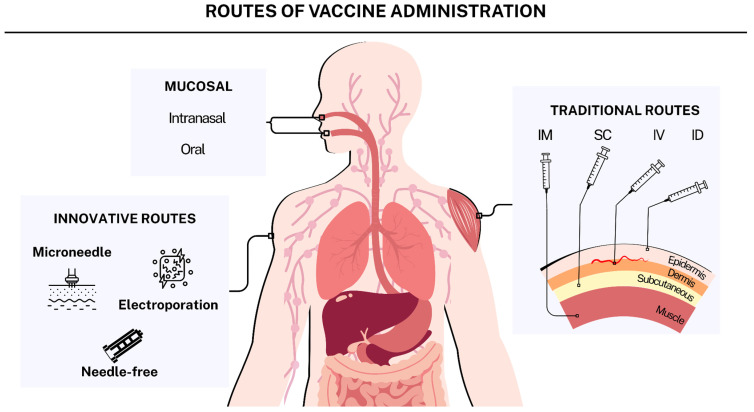
Vaccine administration routes. Schematic representation of the different vaccine administration routes, including mucosal (intranasal and oral), traditional (intramuscular—IM, subcutaneous—SC, intravenous—IV, and intradermal—ID), and innovative (microneedle, electroporation, and needle-free).

The intramuscular (IM) route is preferably chosen given its wide distribution of use and relative acceptance, especially when choosing appropriate needles and the use of good techniques are implemented (Figure 4) [316,317]. Still, the subcutaneous (SC) and intradermal (ID) routes are also continuously explored, with vaccines such as BCG (intradermal) and MMR (subcutaneous) used worldwide for disease prevention [318,319]. However, there are concerns about the use of these routes due to local side effects and lower immunogenicity compared to the IM administration [320,321]. Nevertheless, due to the diversity of immune cell populations present in the epithelial tissue, it remains of interest for vaccination purposes [322]. The use of hypodermic syringes and needles, whether short or standard, requires handling by adequately trained professionals. Even so, reports of rejection by patients, poor administration, and adverse effects highlight the importance of systems that mitigate these situations.

Microneedle systems are composed of microscopic-length needles, mostly up to 1000 μm [323], organized in small patches [324], and can be made in solid [325,326], hollow [327,328,329], coated [330,331,332], dissolvable [333,334,335], and hydrogel forms [336,337,338,339]. They are interesting resources in this context, given that vaccine administration with microneedles is often painless as it reaches the superficial layers of the epithelial tissue [324,340]. On the other hand, needle-free vaccinations employ methods and formulations that allow for the administration of vaccines with minimal invasion to the body while aiming to maintain the immunogenicity of the chosen antigens, such as high-pressure fluid injectors [341,342,343,344], biolistics [345,346,347], and sonoporation [348,349].

ZyCoV-D, the first DNA vaccine to be licensed for human use, was granted emergency authorization in India for prophylaxis against SARS-CoV-2 and is being investigated in preclinical and clinical studies and is administered intradermally through a needle-free system, although its results indicate moderate efficacy [350,351,352]. Recently, Generotti and collaborators (2023) [353] evaluated a new combined technique of electroporation (EP) with needle-free ID administration, applying a local vacuum named noninvasive intradermal vacuum-EP (ID-VEP) [353]. In this study, the use of ID-VEP generated a more robust humoral response after vaccination against the Middle East Respiratory Syndrome Coronavirus (MERS-CoV) when compared to ID injection or vacuum application alone [353]. The use of the nasal mucosa for the delivery of vaccines and adjuvants has been studied for the prophylaxis of respiratory viruses, aiming to elicit responses at local sites for the immunological maintenance of the respiratory tract [354]. Some of these employ needle-free methods such as liquid sprays, a method that accounts for most clinical studies [355,356,357,358,359,360]; powder delivery, whose potential for prolonged storage makes it quite interesting, but strategies to increase absorption may be required [361,362,363,364,365]; and mucoadhesive formulations [366,367,368,369,370]. FluMist^®^ is a live attenuated influenza vaccine administered through a nasal spray, licensed since 2003 [359,371], and was recently authorized for self-administration by the U.S. Food and Drug Administration (FDA, 2024 [372]). This highlights the potential of similar methods to popularize immunizations for the prevention of seasonal viruses, as they eliminate the need for frequent visits to medical care centers.

Electroporation involves the application of short pulses of electric current as a means to enhance the cellular absorption of molecules such as DNA, RNA, and drugs [373,374,375,376,377] due to the increase in the permeability of cell membranes [378], and is also associated with additional effects such as the recruitment and prolongation of components of the immune response [373,379,380,381]. In the context of vaccines, EP is extensively explored to enhance responses to DNA vaccines, which, although they can be rapidly produced and stored at room temperature [382], have shown low immunogenicity in clinical studies, partly due to the low entry of these molecules into the cell nucleus, an event necessary for their biological expression [383,384]. Clinical trials are currently underway to test the use of EP in conjunction with vaccination in humans. Some of these were conducted for the evaluation of prophylactic vaccines for Zika [385], Ebola (Tebas et al., 2019 [386]), and HIV [387,388,389]. INO-4800 is a DNA vaccine encoding the S protein of SARS-CoV-2 that was initially evaluated in mice and guinea pigs [390]. When administered via ID injection followed by EP in humans, it was shown to be tolerable and capable of eliciting cellular and humoral responses against SARS-CoV-2 [391], with a subsequent study demonstrating that the antibodies produced in response to the vaccine had neutralizing activity against the global viral variants of the time [392], with the detection of neutralizing antibodies 6 months after the second dose, which could be further prolonged by a booster dose [393]. And although these results cannot be credited to the use of ID/EP, it was shown to be an effective form of antigen delivery.

VGX-3100, a DNA-based immunotherapy composed of two plasmids encoding the E6 and E7 genes of modified HPVs 16 and 18 [394] (PMID: 23052295), was evaluated in patients with cervical lesions [394,395], showing consistent cellular and humoral responses, and, in particular, those which can be attributed to the activity of cytotoxic T lymphocytes and lytic proteins [396], with over 90% of the patients experiencing sustained regression of lesions for 6 months after treatment and a lasting immune response after 18 months [397]. The use of negative pressure at the site after ID injection was studied as a non-harmful alternative to increase the absorption of DNA vaccines, demonstrating that a single dose of a synthetic DNA vaccine against SARS-CoV-2 with suction application had immunogenic activity comparable to two doses of the same vaccine without suction, indicating the potential of this technique in strategies involving the delivery of DNA molecules [398].

Meanwhile, new electroporation devices are being developed, such as the ePatch, an electroporator coupled with a microneedle patch for the application of compounds [399], previously evaluated in mice vaccinated against SARS-CoV-2; its use is linked to an interesting approximate ten-fold increase in antibody titers compared to ID and IM administration without EP [400]. The development of easy-to-handle and portable equipment is something to be considered for the application of EP in large-scale vaccinations. In this sense, microneedle delivery methods, needle-free technologies, and electroporation have shown promise in improving antigen delivery and immune responses. However, their implementation is also related to biological, structural, and logistical constraints, such as population acceptance, cost, and infrastructure. Thus, addressing these translational barriers will be necessary to close the gap between preclinical success and practical application.

## 5. Clinical Applications and Translational Challenges

Despite the evolution in the design of delivery systems and the variety of functionalized particles, some challenges must be overcome for clinical translation to become viable. These challenges involve biological, structural, and logistical issues. Logistics also includes feasibility studies to distribute these products, which is also related to the scaling of vaccine production using functionalized delivery vectors. Furthermore, it should be considered that depending on the functionalization applied to the nanoparticles, the cost of the vaccine product may increase, in addition to imposing specific infrastructure requirements for storage, such as cold chain networks, which limit the broad distribution of vaccine products.

The experimental design of the preclinical phase also impacts further results from clinical studies. Although mice are the main animal models for vaccine studies, due to their physiological similarities and clinical correlation, they may differ from humans in terms of immune response and cellular profiles, including the expression of some receptors [401]. Thus, if the functionalization is structured so that the delivery system interacts with a set of receptors, it is necessary to previously study whether the chosen animal models present a pattern that correlates with the profile present in humans when subjected to vaccination, immunological challenge, or induction of disease/infection. This observation is relevant to evaluating the delivery system and to enabling clinical research. Hence, in some cases, there is a need to use humanized transgenic animal models [402].

It is worth noting that the difficulties and challenges for the clinical application of this approach differ when it comes to the clinical translation of prophylactic and therapeutic vaccines. The functionalization of particles used in delivery systems has the intrinsic advantage of increasing the specificity of vaccine targeting, and this property is crucial for therapeutic vaccines [403]. Some functionalization approaches include the addition of ligands for receptors that have their expression increased in some pathologies, such as in some types of cancer [404,405]. However, despite the patterns associated with these diseases, individual or population variations should be considered for immunotherapy to be truly effective and specific [406].

These differences between prophylactic and therapeutic approaches may be associated with the choice of the agent used for functionalization. As observed in Table 2, there are several ongoing clinical studies employing functionalized particles, with an emphasis on ferritin, which are generally studies aimed at prophylactic vaccines against viruses such as Influenza, EBV, and SARS-CoV-2 [68]. On the other hand, a range of therapeutic vaccines that act as immunotherapies for various types of cancer have been using customized liposomes with DOTAP [407]. In general, acceptable levels of safety have been observed in ongoing phase I/II clinical trials. Some components used for functionalization have shown increasing application in preclinical studies but have not yet reached the clinical phases, such as cell-penetrating peptides. Despite the promising results in studies using CPPs, their mechanisms of action are still unclear, especially concerning cellular uptake, in addition to limitations regarding specificity and stability [408]. Therefore, there is still a need for investment in in vitro and preclinical studies that can help overcome these issues.

## 6. Conclusions

Advances in the functionalization of delivery systems have gained prominence in the rational design of vaccines in order to impact both the prevention and treatment of diseases. Specialized ligands such as mannose, penetrating peptides, and molecular adjuvants emerge as viable alternatives to increase immunological efficacy. Furthermore, biomimetic and microorganism-based systems, combined with alternative methods of vaccine administration, such as microneedles and needle-free systems, offer less invasive approaches to stimulate local immune responses. However, taking the knowledge of ligands and methods for reformulating delivery systems obtained in basic research to clinical application is a path fraught with challenges that require extensive optimization efforts. The complexity of some of these methods, as well as the optimization of the physicochemical properties of the systems, can increase production costs and require specialized infrastructure. Thus, future research should focus on the development of carriers that are multifunctional, combining protection, cell targeting, and activation of the immune response. From our perspective, modularity, scalability, and customization should be prioritized to allow the system to be adapted to different disease contexts and population needs. Despite the obstacles, the encouraging results of ongoing phase I/II clinical trials emphasize the potential of functionalized delivery methods, notably in terms of safety and immunological performance. Ultimately, measures to address technical, regulatory, and logistical challenges could result in the approval of new vaccines and therapies based on functionalized delivery systems.

## Figures and Tables

**Table 1:** Advantages and disadvantages of lipid, nucleic acid, and polymer ligands in vaccine delivery systems.

Class of Biomolecule	Ligand	Example	Advantages	Disadvantages	References
Lipids	Ionizable lipids	DLin-MC3-DMA	High delivery efficiency of siRNA/mRNA; stability at physiological pH.	Potential toxicity due to the slow degradation of the lipid tail.	[95,96]
		SM-102	Used in mRNA vaccines (Moderna); high delivery efficacy and long-term stability.	Pro-inflammatory nature and reactogenicity.	[97,98]
		ALC-0315	Component of the Pfizer/BioNTech vaccine; proven efficacy in mRNA vaccines.	Temperature-sensitive; requires ultra-cold storage.	[95,99]
		C12-200	High transfection efficiency; used in mRNA formulations.	It may be less stable under physiological conditions.	[96]
	Cationic Lipids	DOTMA	High transfection efficiency; used in gene therapy.	Potential cellular toxicity at high concentrations.	[100,101]
		DOTAP	Widely used in cationic liposomes; stability.	It can induce inflammatory responses.	[102,103,104]
		DODAC	Efficient in DNA delivery; stability in formulations.	Less studied compared to DOTAP and DOTMA.	[105]
	Helper Lipids	DSPC	Stabilizes the outer layer of LNPs; improves nanoparticle formation.	It can reduce delivery efficiency in some formulations.	[100,106,107]
		DOPE	Facilitates membrane fusion; improves endosomal release.	It may be less stable in complex formulations.	[108,109]
		Cholesterol	Increases the stability of LNPs; improves cargo delivery.	In excess, it can reduce transfection efficiency.	[110,111,112]
		DMG-PEG2000	Reduces particle aggregation; prolongs circulation time.	It can induce the production of anti-PEG antibodies after repeated doses.	[104,113]
	Adjuvant lipids	C12-TLRa	Activation of TLR7/8; increase in the immunogenicity of mRNA vaccines.	May require dosage optimization to avoid excessive inflammatory reactions.	[97]
	Cholesterol substitutes	Corosolic acid (CA)	Improves cellular penetration; stabilizes nanoparticles.	Fewer clinical studies compared to traditional cholesterol.	[114]
	Modified lipids	DOG-IM4	Greater thermal stability; efficacy in mRNA vaccines.	Complex synthesis; high cost.	[115]
Nucleic Acids	TLR agonists	CpG ODN (TLR9)	Robust activation of the innate immune response; synergy with other adjuvants.	It can induce excessive inflammatory responses at high doses.	[116,117]
	STING agonists	cGAMP	Activation of the STING pathway; potent antitumor responses.	It can be unstable in certain formulations.	[118]
	Synthetic RNA analog	Poly (I:C)	Potent immune activator; mimics viral RNA, stimulating innate immunity.	Can cause excessive immune activation, leading to toxicity or autoimmune responses.	[119,120]
Polymers	PLGA	PLGA-chitosan	Biocompatibility and controlled release of antigens.	It can be quickly eliminated by the immune system.	[121]
	Polyethylenimine (PEI)	PEI-MM-PLGA-DP/OVA	High efficiency of cellular internalization and improvement in the immune response.	Potential toxicity at high concentrations.	[122]
	Polyethylene glycol (PEG)	PEG-DMG	Increases circulation time; reduces particle aggregation.	It can induce the production of anti-PEG antibodies after repeated doses.	[113,123]

**Table 2:** Main clinical phase vaccine studies using functionalized nanoparticles.

Functionalization	Purpose	Status	ID
DOTAP	Novel RNA-nanoparticle vaccine for the treatment of early melanoma recurrence	Phase I (Suspended)	NCT05264974
DOTAP	Escalating dose study to evaluate the safety, tolerability, and pharmacodynamics of a therapeutic vaccine against HPV-positive cervical intraepithelial neoplasia	Phase I (Completed)	NCT02065973
DOTAP	Study of an approach with combination immunotherapy in subjects with advanced HPV-associated malignancies	Phase I/II (Active, not recruiting)	NCT04287868
DOTAP	Demonstration of the manufacturing feasibility and safety and determination of the maximum tolerated dose of RNA-LP vaccines for pediatric high-grade gliomas and adult glioblastoma	Phase I/II (Active, not recruiting)	NCT04573140
Ferritin	Evaluation of the safety of and immune response of a vaccine against EBV	Phase I (Active, not recruiting)	NCT04645147
Ferritin	Evaluation of the safety, reactogenicity, and immune response of a vaccine for COVID-19	Unknown status	NCT04784767
Ferritin	Dose setting and evaluation of the safety, tolerability, and immunogenicity of an influenza vaccine	Phase I (Completed)	NCT03814720; NCT03186781
Matrix-MTM	Assessment of the safety and effectiveness of experimental malaria vaccines	Phase I/IIa (Active, not recruiting)	NCT05978037
PEG-oxime linker	Evaluation of the safety and immunogenicity of a vaccine against respiratory syncytial virus	Phase I (Completed)	NCT04519073
PLGA	Determination of therapeutic vaccine against melanoma	Phase I (Active, not recruiting)	NCT01753089
Mannose	Dosage studies of a mannose receptor-targeted hCG-β vaccine in patients with incurable locally advanced or metastatic breast, colorectal, pancreatic, bladder, and ovarian cancer	Phase I (Completed)	NCT00648102
Mannose	Evaluation of the safety of HIV-1 gp120 C4-V3 hybrid polyvalent peptide immunogen formulated in mineral oil containing mannose mono-oleate in HIV-1 uninfected volunteers	Phase I (Completed)	NCT00000886
β-Glucan	Testing the potential of the allogeneic cellular vaccine 1650-G vaccine to enhance immune recognition of tumor cells in patients with lung cancer	Phase II (Completed)	NCT01829373
β-Glucan	Investigation of the combination of a bivalent vaccine, β-glucan, and GM-CSF as an effective treatment for people with high-risk neuroblastoma	Phase II (Recruiting)	NCT04936529
β-Glucan	Study about the efficacy and tolerability of β1,3/1,6-glucan with inactivated *Saccharomyces cerevisiae* rich in selenium and zinc in volunteers receiving influenza or COVID-19 vaccine	Completed	NCT04798677

## Abbreviations

The following abbreviations are used in this manuscript:

ADM	allyl-α-D-mannopyranoside
ALC-0315	ionizable lipid used in Pfizer-BioNTech’s COVID-19 vaccine
AMA-1	Apical Membrane Antigen-1
APC	antigen-presenting cell
BCG	Bacille Calmette-Guérin
BG	bacterial ghost
BMV	bacterial membrane vesicle
cGAMP	cyclic GMP-AMP (STING agonist)
CLR	C-type Lectin Receptor
COE	neutralizing epitope of porcine epidemic diarrhea virus (PEDV)
CPP	cell-penetrating peptide
CTL	cytotoxic T lymphocyte
DC	dendritic cell
DC-SIGN	Dendritic Cell-Specific Intercellular Adhesion Molecule-3
Dex	Dendritic Cell-derived Exosomes
DOPE	1,2-Dioleoyl-sn-glycero-3-phosphoethanolamine
DOTAP	1,2-Dioleoyl-3-trimethylammonium-propane
DOTMA	N-[1-(2,3-Dioleoyloxy)propyl]-N,N,N-trimethylammonium chloride
EP	electroporation
FMDV	foot-and-mouth disease virus
GAS	Group A streptococcus
GP	glucan particle
GPI	glycosyl-phosphatidylinositol
GRAS	generally recognized as safe
HA	hemagglutinin
HLA	Human Leukocyte Antigen
ID	intradermal
IM	intramuscular
LNP	lipid nanoparticle
LSCExo	lung cell-derived exosome
MHC	major histocompatibility complex
MR	mannose receptor
mRNA	Messenger RNA
MeNPs	metallic nanoparticles
MOFs	metal–organic frameworks
MUC1	Mucin 1
PAMP	pathogen-associated molecular pattern
PBAE	poly(β-amino ester)
PEDV	porcine epidemic diarrhea virus
PEG	polyethylene glycol
PEI	polyethylenimine
PLGA	poly(lactic-co-glycolic acid)
PLPs	phage-like particles
PNP	polymeric nanoparticle
PRR	pattern recognition receptor
RABV	rabies virus
RBD	receptor-binding domain
RGD	arginine–glycine–aspartic acid (integrin-binding peptide)
SC	subcutaneous
siRNA	Small Interfering RNA
SM-102	ionizable lipid used in Moderna’s COVID-19 vaccine
STING	Stimulator of Interferon Genes
TLR	Toll-like receptor
TNBC	triple-negative breast cancer
WYV	whole yeast vaccine
YSD	yeast surface display
YS	yeast shell
